# Reply to ‘Misestimation of heritability and prediction accuracy of male-pattern baldness’

**DOI:** 10.1038/s41467-018-04808-2

**Published:** 2018-06-29

**Authors:** Nicola Pirastu, Peter K. Joshi, Paul S. de Vries, Marilyn C. Cornelis, NaNa Keum, Nora Franceschini, Marco Colombo, Edward L. Giovannucci, Athina Spiliopoulou, Lude Franke, Kari E. North, Peter Kraft, Alanna C. Morrison, Tõnu Esko, James F. Wilson

**Affiliations:** 1 Centre for Global Health Research, Usher Institute of Population Health Sciences and Informatics, University of Edinburgh, Teviot Place, Edinburgh, EH8 9AG UK; 2 Human Genetics Center, University of Texas Health Science Center at Houston, Houston, TX 77030 USA; 3 Department of Preventive Medicine, Northwestern University Feinberg School of Medicine, Chicago, IL 60611 USA; 4 Department of Nutrition, Harvard T. H. Chan School of Public Health, Boston, MA 02115 USA; 5 Department of Epidemiology and Carolina Center for Genome Sciences, University of North Carolina, Chapel Hill, NC 27599 USA; 6 Center for Population Health Sciences, Usher Institute of Population Health Sciences and Informatics, University of Edinburgh, Teviot Place, Edinburgh, EH8 9AG UK; 7 Department of Epidemiology, Harvard T. H. Chan School of Public Health, Boston, MA 0211 USA; 8 Channing Division of Network Medicine, Department of Medicine, Brigham and Women’s Hospital and Harvard Medical School, Boston, MA 02115 USA; 9 Pharmatics Ltd, Edinburgh, EH16 4UX UK; 10 Department of Genetics, University Medical Center, 9713 GZ Gröningen, The Netherlands; 11 Program in Genetic Epidemiology and Statistical Genetics, Harvard T. H. Chan School of Public Health, Boston, MA 02115 USA; 12 Estonian Genome Center, University of Tartu, 51010 Tartu, Estonia; 13 Broad Institute of Harvard and MIT, Cambridge, MA 02142 USA; 14 MRC Human Genetics Unit, Institute of Genetics and Molecular Medicine, University of Edinburgh, Western General Hospital, Crewe Road, Edinburgh, EH4 2XU UK

Yap et al.^1^ present two criticisms of our recent analysis^2^ of the genetic architecture of male pattern baldness (MPB)^2^. First they note our earlier study in ref.^2^ overestimated SNP heritability (hereafter heritability) by excluding people in category two on the UK Biobank scale. We agree, heritability should have been reported as 0.64 not 0.94. This arose for a natural, if mistaken for this purpose, desire to categorize subjects clearly on a binary scale, and thus exclude indeterminate subjects. Their second criticism is that we overestimated the proportion of heritability explained by the 71 loci that have been identified. We stand by the broad conclusion that about one-third of the genetic effects (on a baldness trait dichotomized as category 1 versus 2, 3, or 4) are explained by the 71-locus SNP score.

In principle, the proportion of polygenic variance explained by the SNP score can be evaluated in the following three possible ways: (1) as the ratio of the phenotypic variance explained by the SNP score to the variance explained by polygenic effects; (2) as the ratio between the heritability due to the SNPs and the baseline heritability estimate; or (3) as ratio of the reduction in polygenic variance in a model that includes the SNP score to the polygenic variance in a model that does not include this fixed effect (see Supplementary Method for details). These three methods should give the same result if the residual variance and the phenotypic variance do not change between the models with and without the SNPs.

Yap et al. have used the first method, and estimate that the 107 SNPs from 71 loci explain about 15–20% of variation in total liability. Our own estimate using the same method is 20% on the liability scale, close to theirs, implying that about 31% of the total heritability of 0.61 is explained by the SNP score. Our article, however, reported an estimate by method (2), in which the ratio of the difference in heritability in models including and excluding the SNP to the baseline heritability was 38%. Including category two did not change this estimate (Supplementary Table 1 method (2)). Of course, to evaluate predictive performance requires an independent test dataset, beyond the scope of both our original study^2^ and the correspondence^1^.

GCTA implements a mixed linear model and therefore estimates phenotypic variance from the variances of the random effects in the model. Therefore, the estimated phenotypic variances from models with different fixed effects (i.e., with and without the SNP predictor) are different. We thus applied method (3) which is not affected by the same issue as it works on the absolute and not relative scale (see Supplementary Method) and gives an even greater estimate of 45%. Given the limitations of fitting mixed linear models to a binary trait to estimate the parameter of interest, it is not easy to be certain which is the best one. However, irrespective of which is used, our conclusion that we can explain a relatively large proportion of heritability using SNPs from only 71 loci is still valid.

Having now corrected the error in the estimation of heritability, we thus believe that the remainder of the results and conclusions are still valid, including in particular that we can explain a large proportion of the genetic variance using a relatively small number of SNPs. Furthermore, our identification and replication of several new loci for MBP remains accurate and increases the understanding and biological interpretation whilst highlighting the shared genetics with other traits.

## Data availability

All data that support the findings of this study are available from the corresponding author upon reasonable request.

## Electronic supplementary material

Supplementary Information
