# Supplementary material for: Reply to ‘Misestimation of heritability and prediction accuracy of male-pattern baldness’
Source: Nat Commun. 2018 Jun 29;9:2538. doi: 10.1038/s41467-018-04808-2 (PMC6026187; doi:10.1038/s41467-018-04808-2)
Supplement: Supplementary file 1 — Supplementary Information [file 41467_2018_4808_MOESM1_ESM.pdf]

Supplementary Information

Supplementary Method

It is important to note that in the case of proportion of heritability, the scale of estimation (liability or observed) does not matter, in fact according to formula [1] in Yap et al. the transformation depends only on the denominator, which is a constant that depends only on the population composition and thus will not affect the proportion since multiplying the numerator and the denominator for a constant value will give the same result.

If we denote $V(g_0)$ as the variance explained by the polygenic effect, which corresponds to the additive genetic variance, $V(p_0)$ the trait variance, $V(g_1)$ the residual variance explained by the polygenic effect after including the SNPs as fixed effects and $V(p_1)$ the corresponding residual phenotypic variance, and if we assume the SNPs will affect V(p) only through V(g), then the proportion of genetic variance explained by the SNPs can be easily computed as:

$(V(p_0)-V(p_1))/V(g_0)$ (1)

Or as:

$(h^2_0-h^2_1)/h^2_0$ (2)

Or as:

$(V(g_0)-V(g_1))/V(g_0)$ (3)

If the difference in V(p) was due only to the effect of the SNPs and if h^2_0 and h^2_1 refer to the proportions of total phenotypic variance $V(p_0)$ due to the polygenic effects $V(g_0)$ and $V(g_1)$, respectively, thus dividing all terms by the same number, we should obtain the same result using (1), (2) or (3).

Hence, we first estimated the heritability using only the base model which included age, 10 PCs, center of assessment and array batch, we then computed a second estimate of the heritability including the 107 SNPs as fixed effects. We then used (2) to estimate the proportion of heritability explained which gave 38% in our original paper. In order to verify if the exclusion of category two influenced this value, we re-estimated this proportion on 20,000 different random samples including this time also the people in category 2. Using the same method (2), we still obtained 38%.

The problem in this approach is that the heritability from the two models is estimated from different phenotypic variances, because variation due to fixed effects does not contribute to the estimated variance components. We realised this only after the correspondence by Yap et al[1]. For this reason we can't directly use (2) as we did, instead of (3) since the phenotypic variances to which the two heritability refer to are different.

	Baseline model	Model with SNPs as fixed effects	Difference in genetic variance	Proportion of additive genetic variance explained by the SNPs $(V(g_0)-V(g_1))/V(g_0)$	Estimation method
**V(autosomes)**	0.063407	0.038894	0.024513	0.3865977	
**V(X chromosome)**	0.013699	0.003040	0.010659	0.778086	
**V(g)**	0.077106	0.041934	0.035172	0.4561513	(3)
**V(e)**	0.136316	0.147085			
**V(p)**	0.213422	0.189019	0.024403	0.3164864	(1)
**h_o^2 (V(g)/V(p))**	0.361284	0.2218507	0.1394335	0.3859385	(2)
**h_l^2**	0.616826	0.3787695	0.2380572	0.3859385	(2)

Supplementary Table 1 Variance components on the absolute scale including all baldness categories. Calculations were done in GCTA. V(g) is the genetic variance, V(e) the environmental variance, V(p) the phenotypic variance, V(autosomes) the autosomal genetic variance, V(X chromosome) the X chromosomal genetic variance. The three different estimation methods used are specified in the last column. h_o^2 reports the heritabilities on the observed scale while h_l^2 reports the same estimate transformed to the liability scale.
The values in the V(p) row are analogous to the method used by Yap et al. to estimate the proportion of explained variance. There is a large difference between estimating the difference in variance using the (1) and (3) due to the increase of the residual variance.
